# Detection of Bovine Viral Diarrhoea Virus in a Case Series of Clinically Cachectic Cattle from Tiaret, Algeria

**DOI:** 10.3390/vetsci12121193

**Published:** 2025-12-12

**Authors:** Nacira Ghenoumat, Houari Hemida, Assia Boumezrag, Dimitrije Glišić, Sofija Šolaja, Ljubiša Veljović, Vesna Milićević

**Affiliations:** 1 Laboratory for Improvement and Valorisation of Local Animal Production, Institute of Veterinary Sciences, University of Tiaret, Tiaret 14000, Algeria; hemida.houari@univ-tiaret.dz (H.H.);; 2 Virology Department, Institute of Veterinary Medicine of Serbia, Janisa Janulisa 14, 11000 Belgrade, Serbiavesna.milicevic@nivs.rs (V.M.)

**Keywords:** BVDV, cachexia, cattle, immunosuppression, seropositive, BVDV-1f, secondary infection

## Abstract

Bovine viral diarrhea virus (BVDV) poses a serious disease in cattle. Cachexia is a condition marked by weight loss and muscle wasting and is often observed in infected animals. This study reports a case series from Tiaret province, Algeria, aimed at detecting and genetically identifying BVDV in clinically cachectic cattle. A total of 100 cachectic animals from ten herds were examined using serological, molecular, and virological methods, including antibody ELISA, RT-qPCR, and virus isolation in cell culture. Overall, 93.6% of the animals tested were seropositive for BVDV, indicating extensive exposure to the sampled population. Higher antibody rates were observed in adults (≥12 months) and males compared to younger animals and females, but the differences were not statistically significant. Phylogenetic analysis of the 5′UTR sequences confirmed the presence of the BVDV-1f subtype closely related to sequences reported in Europe. Although BVDV was detected in animals within the sampled herds, the data does not support a causal conclusion that BVDV was the direct cause of cachexia in this case series; rather, its immunosuppressive effects may predispose cattle to secondary infections contributing to wasting. These results are specific to this opportunistic case series from Tiaret province and should be considered exploratory; broader and representative surveillance is required to assess the national epidemiological situation.

## Simple Summary

Bovine viral diarrhoea (BVD) is an important infectious disease of cattle that causes health and economic problems. This report describes the detection and genetic identification of BVDV-1f in a clinical series of cachectic cattle from Tiaret province, Algeria. One hundred clinically cachectic cattle from ten herds were sampled opportunistically and tested using serological and molecular methods. Molecular analysis identified a BVDV-1f lineage closely related to sequences previously reported in Europe. While BVDV was common among the sampled animals, the virus does not appear to be the sole cause of severe weight loss; instead, its immunosuppressive effects may predispose animals to other diseases that contribute to cachexia. These findings provide local baseline data and highlight the need for broader, representative surveillance to determine BVDV’s epidemiological role in Algeria.

## 1. Introduction

Bovine viral diarrhoea (BVD) is a serious threat to cattle worldwide [1]. The responsible pathogen comprises 19 recognized species; only three are of primary relevance to cattle: Pestivirus A (BVDV-1), Pestivirus B (BVDV-2) and Pestivirus H (HoBi-like pestivirus). These enveloped, positive-sense, single-stranded RNA viruses that fall under the genus Pestivirus within the Flaviviridae family [2], contain one large open reading frame (ORF) that encodes four structural and eight non-structural proteins, flanked by 5′ and 3′ untranslated regions [3]. To date, researchers have identified 24 BVDV-1 subtypes (ranging from 1a to 1x) and 5 BVDV-2 subtypes (2a to 2e) [4]. Given the limited availability of complete genome data, genotyping relies primarily on 5′-UTR sequences available in public databases [5]. BVDV occurs as two biological biotypes: non-cytopathic (NCP) and cytopathic (CP). Infection of seronegative pregnant cows with NCP strains during early gestation (~40–120 days) can produce persistently infected (PI) offspring that shed virus lifelong and act as major reservoirs within herds; CP strains generally arise by mutation of NCP viruses and are associated with mucosal disease [6,7]. Transmission is through direct contact and indirect exposure through contaminated surfaces, feed, aerosols, and fomites [8,9]. Infection may lead to clinical manifestations, that vary from subclinical to acute haemorrhagic syndrome and lethal mucosal disease. BVDV induces immunosuppression, which aggravates clinical signs, particularly in the presence of concurrent infections [10].

In Algeria, the national herd was estimated at approximately 1.78 million cattle in 2019 [11]. Tiaret province accounted for 49,230 animals in 2019, representing about 2.8% of the national herd [12]. Currently, approximately 96% of Algerian farms raise imported cattle; Prim’ Holstein and Pie-Rouge des Plaines represent about 75% and 20% of the national herd, respectively [13,14,15]. Recent surveys showed that, poor body condition is common with: 35.1% of cows classified as cachectic (body condition score (BCS) ≤ 2 on a 0–5 scale) [16]. Evidence from other regions supports a link between BCS and BVDV exposure; for example, a large cross-sectional study in Bangladesh reported that thin cows had markedly higher odds of BVDV seropositivity [17]. Taken together, these observations motivate targeted investigation of BVDV circulation in cachectic animals in Tiaret province and the potential contribution of infection to poor body condition; broader studies are required to extrapolate findings beyond this province.

Several studies, including [18], have reported BVDV circulation and described its epidemiological patterns among different animal populations in Algeria. This exploratory case series aimed to detect and genetically type BVDV in clinically cachectic cattle from Tiaret province, Algeria. Using complementary serological, molecular, and virological approaches, we sought to confirm BVDV infection and identify circulating viral types within the examined population.

## 2. Materials and Methods

### 2.1. Animals

This study involved 100 cachectic cattle of both sexes, from 10 herds (Table 1). The animals included local, imported, and predominantly crossbreeds from Tiaret province in northwestern Algeria. Sampling focused on the cachectic subgroup within each herd, regardless of herd size. Animals were selected based on clinical evaluation in ten herds. Cachexia was identified by a poor body condition score of ≤2 on a 0 to 5 scale as previously described [19]. This study used opportunistic convenience sampling of clinically cachectic animals within Tiaret province and therefore does not represent a randomized or nationwide survey.

### 2.2. Blood Sample Collection

The study was conducted in Tiaret Province (35°22′ N, 1°20′ E), Algeria, located approximately 160 km south of the Mediterranean coast and covering an area of about 20,400 km^2^. A total of 100 blood samples were collected from cattle (Table 1). After collection, the samples were delivered to the Institute of Veterinary Sciences in Tiaret, where serum separation was performed by centrifugation at 1500 g for 10 min. The recovered serum was decanted and stored at –20 °C. The samples were then transported in an icebox to the Institute of Veterinary Medicine in Belgrade, Serbia, for molecular and serological analyses, following international regulations for the transport of biological materials. All 100 samples were prioritised for nucleic acid extraction and RT-qPCR. During processing, six samples yielded insufficient serum volume to run the antibody ELISA, therefore ELISA was performed on 94 sera only.

### 2.3. Enzyme-Linked Immunosorbent Assay for BVDV Detection

The commercially available ID Screen^®^ BVD p80 Antibody Competition ELISA kit (Innovative Diagnostics, Grabels, France) was used to detect antibodies against the p80 (NS3) protein in serum samples. The competitive ELISA demonstrated high sensitivity and specificity [20]. The test and result interpretation were performed according to the manufacturer’s instructions. The optical density was measured with a microplate reader (Multiskan MS LabSystems^®^ Microplate Reader, Vantaa, Finland).

The test is considered valid if: The mean value of the negative control (ODNC) is >0.7, the mean value of the positive control (ODPC) is <30% of ODNC. The percentage of competition (S/N%) was calculated using the formula: S/N% = (OD sample/ODNC) × 100. Results interpreted as positive if: S/N% ≤ 40%, doubtful if: 40% < S/N% ≤ 50% and negative if: S/N% > 50%.

### 2.4. RNA Extraction and RT-PCR

Nucleic acid was extracted from 200 µL of each serum sample using the IndiMag^®^ Pathogen Kit (QIAGEN for INDICAL BIOSCIENCE, Leipzig, Germany) with the IndiMag 48s (INDICAL) extraction system. Amplification of viral RNA was conducted using the Luna Universal One-Step RT-qPCR Kit (New England Biolabs^®^ Ipswich, MA, USA) on an Applied Biosystems Quant Studio 3 Real-Time PCR System. Briefly, the amplification was carried out in a 12.5 µL reaction volume assembled on ice (2.5 µL template RNA per sample in combination with 10 µL of Reaction mix, which includes 0.4 µL of each primer (10 µM) and 0.2 µL of probe [21].

The thermal cycling protocol included a single reverse transcription step at 55 °C for 10 min, followed by initial denaturation at 95 °C for 1 min. This was succeeded by 50 amplification cycles consisting of denaturation at 95 °C for 10 s and a combined annealing and extension phase at 60 °C for 30 s. Samples with a Ct value below 45 were classified as positive.

RT-qPCR positive samples were subjected to conventional gel-based RT-PCR aiming to generate amplicons for amplification and sequencing of the 5’UTR region, using QIAGEN One Step RT-PCR Kit (Qiagen, Hilden, Germany) and primers 324-F 5′-ATGCCCWTAGTAGGACTAGCA-3′ and 326-R 5′-TCAACTCCATGTGCCATGTAC-3′, amplifying ~288 bp and 299 bp region [22]. The reaction mixture was consisted of 2 µL of template and 18 µL of master mix (6 µL RNase-free water, 0.8 QIAGEN One Step RT-PCR Enzyme Mix, 4 µL 5x QIAGEN One Step RT-PCR Buffer, 0.8 µL dNTP Mix, 4 µL 5x Q-Solution, 1.2 µL of each primer (10 µM), and 2 µL template). RT-PCR products were analysed in a 2% agarose gel stained with ethidium bromide and visualised under UV light after gel electrophoresis, and PCR products of specific length (263 base pairs) were sent to LGC Germany for sequencing.

### 2.5. Phylogenetic Analysis of BVDV

Phylogenetic analysis was initiated by comparing the attained nucleotide sequences with reference sequences available in GenBank using the Basic Local Alignment Search Tool (BLAST + 2.16.0) to identify closely related strains. Representative sequences showing the highest similarity were selected for inclusion in the analysis. Multiple sequence alignment was performed with ClustalW, and phylogenetic trees were inferred using the Maximum Likelihood (ML) method implemented in MEGA X. The most appropriate nucleotide substitution model was established with the MEGA X Find Best DNA/Protein Models feature, and evolutionary distances were calculated according to the Tamura-Nei model. The reliability of the phylogenetic tree topology was assessed by performing 1000 bootstrap replicates.

### 2.6. Viral Isolation

BVDV isolation from RT-qPCR-positive samples was carried out in Madin-Darby Bovine Kidney (MDBK, ATCC CCL-22) cell culture. The cells were cultured in Gibco DMEM/F-12 GlutaMAX ™ medium (Fisher Scientific, Vantaa, Finland), enriched with 10% foetal bovine serum (FBS) for essential nutrients and MycoZap^®^ (Basel, Switzerland) to avoid contamination. Serum samples (100 µL) were inoculated into an 80% MDBK confluent layer of 24 24-well plate and incubated for 7 days at 37 °C with 5% CO_2_. As BVDV is typically non-cytopathic, Immunoperoxidase Monolayer Assay (IPMA) was conducted on cells after each passage, by the WOAH protocol. Fixation was carried out by incubating the plates at 75 °C for 2.5 h to ensure proper adherence and stabilization of the cell monolayer. For antigen detection, an anti-BVDV monoclonal antibody (BIO 295; Bio-X Diagnostics, Rochefort, Belgium) was employed, followed by a secondary sheep anti-mouse peroxidase-conjugated antibody (BIO 408; Bio-X Diagnostics, Rochefort, Belgium). Samples that tested negative were subjected to additional passages, with a total of three serial passages performed.

### 2.7. Statistical Analysis

Statistical analyses were carried out using R software (version 4.5.1). The proportion of tested animals that were seropositive for BVDV was calculated overall and stratified by age group and sex. The Wilson score method was used to compute 95% confidence intervals for these proportions, and Fisher’s exact test was applied to compare groups. Because sampling targeted stunted and clinically cachectic animals and was not random, these proportions are descriptive of the sampled series. A two-sided *p*-value < 0.05 was considered statistically significant.

## 3. Results

### 3.1. ELISA for BVDV Antibody Detection

Out of 94 serum samples, 88 animals (93.6%) were seropositive for BVDV antibodies, 4 (4.2%) were seronegative, and 2 (2.1%) had doubtful results. For precision estimation, only samples with clear-cut results (positive or negative) were considered. When excluding doubtful samples, the overall proportion of seropositive animals reached 95.7% (95% CI: 89.3–98.3%).

When stratified by sex, the proportion of seropositive animals among those tested was 94.6% (95% CI: 83.8–96.3%) in females and 100% (95% CI: 82.4–100%) in males. The age-based analysis revealed high and comparable seropositivity across all age groups, with 94.7% (CI: 75.4–99.1%) in animals younger than 6 months, 95% (CI: 76.4–99.1%) in those aged 6–12 months, and 96.2% (95% CI: 87.2–99%) in animals older than 12 months (Table 2). No significant differences were observed between sexes (Fisher’s exact test, *p* = 0.582) or among age groups (*p* = 1.000).

### 3.2. RT-qPCR and Sequencing

**Figure 1:**
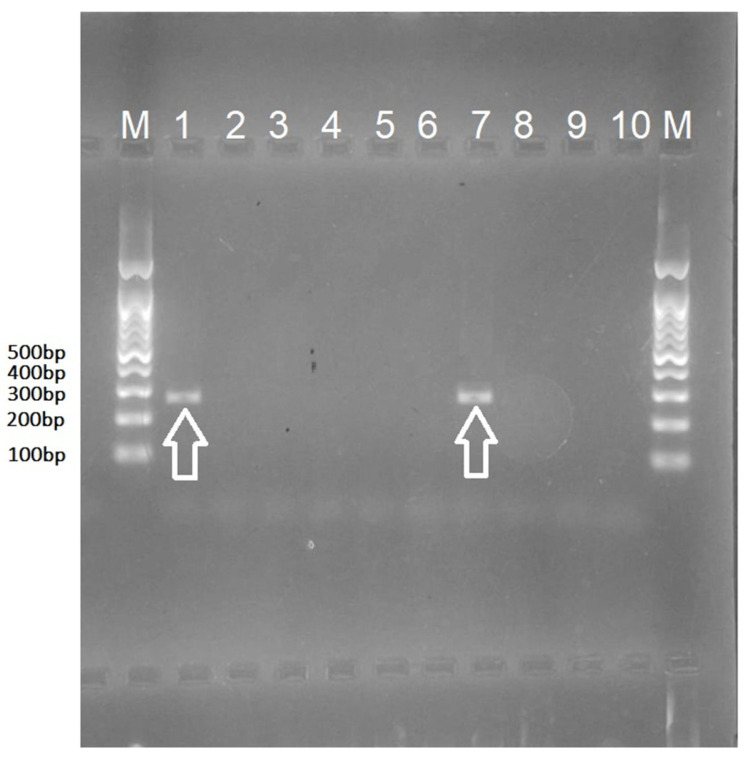
Agarose gel electrophoresis of RT-PCR products targeting the 5′UTR of BVDV. Lane M: 100 bp DNA ladder; lanes 1 and 7 (indicated by arrows) show positive samples; lanes 2–6 and 8–10: negative samples.

**Figure 2:**
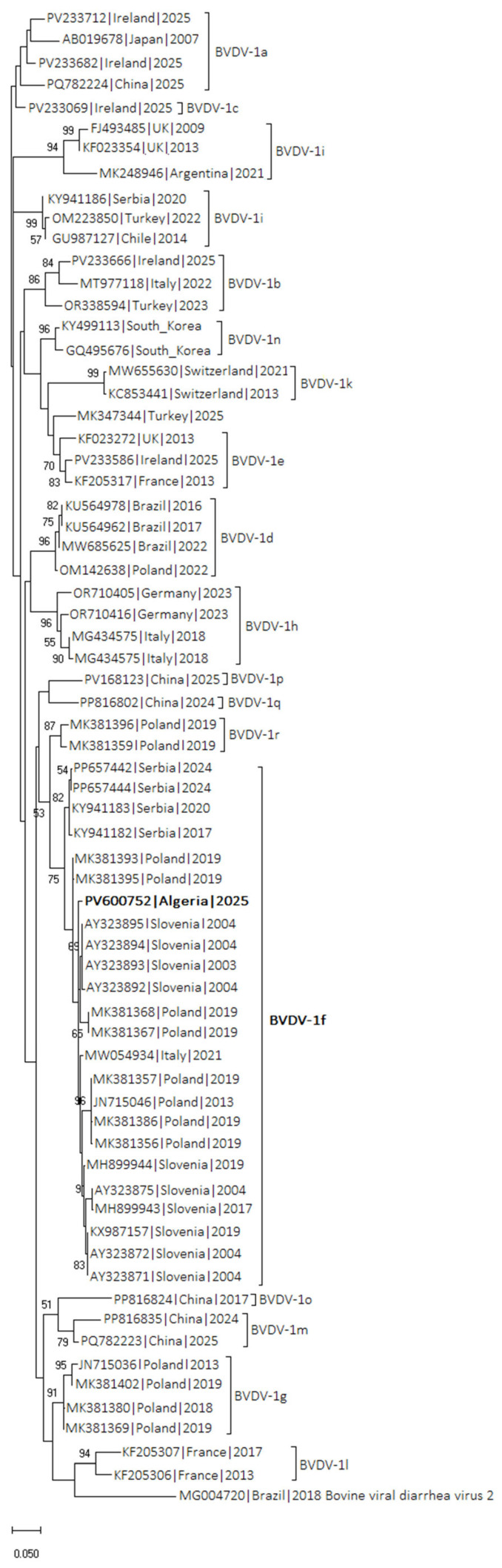
The tree includes bootstrap values based on 1000 replicates, with support values shown next to the branches (only those ≥50% are displayed). Taxa are labelled with their NCBI accession numbers, country of origin, and year of isolation; the sequence obtained in this study is highlighted in bold. Evolutionary analyses were conducted using MEGA X (version 10.2).

Out of 100 tested samples for BVDV, 10 were BVDV-1 positive based on RT-qPCR. The cycle threshold (Ct) values for these positive samples are presented in Supplementary Table S1. Among these, 2 samples exhibited distinct bands on gel-based RT-PCR (Figure 1, original image Figure S1 for details) at the predicted size and were selected for Sanger sequencing. Sequence analysis revealed that the obtained 263 bp 5′UTR sequences shared 100% nucleotide identity; therefore, only one representative sequence was deposited in the NCBI GenBank database under accession number PV600752. Phylogenetic analysis (Figure 2), performed in MEGA X using the Maximum Likelihood method with the Tamura-Nei model and 1000 bootstrap replicates, revealed that the virus belongs to the BVDV-1f subtype. The sequence clustered with high bootstrap support (≥90%) alongside strains MK381368 and MK381367 from Poland and Slovenia. Bootstrap support values were shown next to the branches, with only those ≥50% displayed. These findings confirm the circulation of the BVDV-1f subtype among the sampled animals from Tiaret province, in agreement with nucleotide distance analysis (see Supplementary File S1 Matrix Output).

The figure shows amplification of the 5′UTR region of the BVDV detected by RT-PCR. Positive samples (lanes 1 and 7) showed distinct amplicons of the expected size, whereas no amplification was detected in the negative samples (lanes 2–6 and 8–10). The specific bands corresponding to the expected amplicon size are indicated by arrows, confirming the presence of BVDV RNA in the tested specimens.

### 3.3. Viral Isolation Results

**Figure 3:**
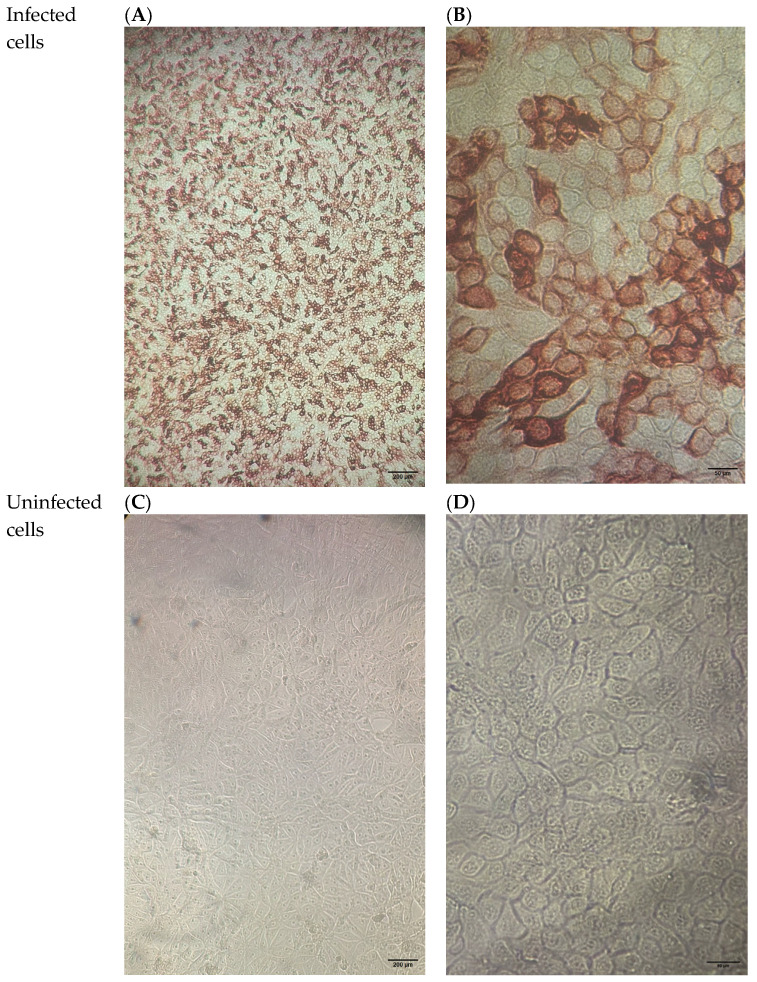
Microscopic images of bovine cells infected with BVDV compared to uninfected controls. Images were taken with a Leica microscope at 100× and 400× magnification, corresponding to approximate fields of view of 2 mm and 0.5 mm, respectively. (**A**) Infected cells at 100× magnification, showing positive immunoperoxidase (IPMA) staining without cytopathic effect. (**B**) Infected cells at 400× magnification. (**C**) Uninfected cells at 100× magnification. (**D**) Uninfected cells at 400× magnification, showing normal morphology.

IPMA revealed specific immunostaining in 2 out of 10 isolates. Notably, no cytopathic effects were observed, confirming the presence of the antigen and the absence of associated cellular damage (Figure 3).

## 4. Discussion

BVDV is a globally distributed pathogen of major significance for cattle health and productivity. Although national surveillance data remain limited, BVDV circulation has been reported across several regions of Algeria [18]. In this cross-sectional case series, we integrated serological, molecular, and virological methods to evaluate BVDV exposure and circulation among clinically cachectic cattle from Tiaret province. The exceptionally high proportion of seropositive animals (93.6%) indicates extensive viral exposure. However, because sampling targeted clinically cachectic animals, these findings cannot alone confirm viral persistence within herds. Longitudinal follow-up is essential to identify PI animals, as their continual presence underpins viral maintenance [23,24]. Conversely, 4.26% of animals were seronegative, suggesting a small naïve subgroup within the tested population.

The high proportion of seropositive animals observed in this study, contrasts sharply with earlier findings about BVDV in Algeria, where Ref. [25], reported only 1.39% These discrepancies can largely be explained by the differences in sampling frame and herd type. Earlier studies often focused on intensively managed dairy herds, whereas our sampling targeted animals in traditional production systems, which present 70% of the total number of farms in the country [26], and are characterised by mixed species grazing, frequent introduction of new stock and minimal biosecurity, tend to facilitate viral exposure and persistence [27]. Guidoum et al. [28] reported a seroprevalence of 59.9% at the same study area, reinforcing evidence that BVDV is enzootic in parts of Algeria.

The high proportion observed across all age groups suggests widespread exposure of animals to BVDV. Although adults exhibited slightly higher antibody prevalence than younger cattle, the overlapping confidence intervals indicate no statistically significant age-related difference. This trend is consistent with cumulative exposure dynamics described in previous studies [29,30,31]. Similarly, all males and 94.6% of females tested seropositive. Although this contrasts with [30], who reported higher prevalence among females, it agrees with previous studies [31,32], that found greater rates in males. This difference could be attributed to increased exposure among breeding males; however, the small number of males sampled might have resulted in an overestimation. Despite these differences, no statistically significant associations with sex or age were detected. BVDV-negative cachectic animals likely suffered from other chronic causes of emaciation, reflecting the multifactorial nature of cachexia [33], including paratuberculosis [34] or parasitic infestations [35].

Combined serological and molecular testing revealed a heterogeneous infection status among the examined cattle. Four animals tested negative in both ELISA and RT-PCR. indicating a naïve immunological profile. According to Ref. [36], such animals have no prior exposure to BVDV, lack both detectable antibodies and viral RNA, and are therefore fully susceptible to infection upon viral introduction. In contrast, seventy-nine animals were ELISA-positive but RT-qPCR–negative, suggesting past exposure and successful viral clearance, with no evidence of active viremia at the time of sampling. This pattern is consistent with resolved or historical infection, where antibodies persist after the virus has been eliminated [36].

Eight animals were double-positive by ELISA and RT-qPCR, with Ct values ranging from 19.7 to 30.6, indicating heterogeneous viral RNA loads among seropositive individuals. Among the ten RT-qPCR positive animals, eight were seropositive, reflecting the simultaneous presence of viral RNA and virus-specific antibodies. Such profiles are typically associated with recent transient infections, in which viremia persists during the early phase of immune response development. In natural infection, these animals are generally undergoing seroconversion while still shedding virus [37]. The detection of viral RNA in adult, seropositive animals may therefore represent short-term viremia following re-exposure or reinfection with antigenically related strains. Similar findings have been reported in highly endemic herds where transient infections can occur even in immunologically experienced cattle [38].

Among the ten RT-qPCR–positive animals, two (ID E9, ELISA doubtful, Ct = 18.4; and ID H14, ELISA negative, Ct = 19.7) from two different herds (Herd 5 and Herd 8) yielded NCP BVDV strains in MDBK cell culture, confirming the presence of replication-competent virus. These Ct values fall within the threshold range associated with successful virus isolation, as reported by Ref. [39] who demonstrated that samples with Ct ≤ 25 consistently yielded infectious virus, whereas those with Ct > 30 rarely did so. This concordance further substantiates the inverse relationship between Ct values and viral infectivity in vitro. The absence of detectable antibodies in animal ID H14, combined with a high viral load and successful virus isolation, provides compelling evidence suggestive of a PI status [40]. Nevertheless, because definitive PI confirmation requires at least two positive RT-qPCR or virus isolation results obtained three or more weeks apart, this classification remains provisional. The eight-month-old calf (ID E9), exhibiting an ELISA-doubtful but RT-qPCR–positive profile with a Ct of 18.4, likely represents a transient infection, potentially reflecting waning maternal antibodies or early seroconversion [13,39]. (see Supplementary Table S1).

The recovery of NCP biotypes from both animals provides evidence of active viral circulation despite the absence or low levels of detectable antibodies, consistent with the predominance of NCP strains under natural field conditions [41]. CP variants typically arise within PI animals through genomic mutation or recombination events. Superinfection of an immunotolerant PI host with both NCP and CP strains triggers mucosal disease, a frequently fatal outcome, while horizontal transmission of CP variants among PI cattle can lead to additional mucosal disease outbreaks within the herd [42]. Maternal antibodies can inhibit viral isolation by binding viral particles and masking epitopes, leading to false-negative outcomes in both culture and antigen ELISA [43,44]

In contrast, RT-PCR is minimally affected by such interference, making it the most reliable diagnostic approach for BVDV detection in colostrum-fed calves [36].

Collectively, these findings underscore the value of integrating serological, molecular, and virological assays to improve diagnostic sensitivity in local surveillance efforts; however, because sampling was opportunistic and limited to Tiaret province, these results should be interpreted as exploratory and not taken as a national prevalence estimate.

BVDV’s immunosuppressive effects may predispose animals to secondary infections or exacerbate existing diseases [10,15], leading to marked weight loss. Our findings indicate a notably high proportion of BVDV-positive animals among the cachectic cattle tested.; however, this represents an association rather than definitive evidence of causation. Cachexia is a complex and multifactorial syndrome, and while BVDV may contribute to weight loss directly through immunosuppression or indirectly by predisposing animals to respiratory and gastrointestinal infections [14], the role of other diseases cannot be excluded.

Our findings match previous studies showing that BVDV is widely distributed across North Africa, though the considerable variability in seroprevalence by country. Elkhoja et al. [30] noted low prevalence in Tunisia (11.8%) and moderate prevalence in Libya (48.6%). Alali et al. [45] reported 56.1% seroprevalence in Morocco. Such heterogeneity would be the result of variation in animal movement patterns, biosecurity deficiencies, and the lack of harmonised control programs. Nevertheless, it confirms that BVDV is endemic throughout North Africa. Reference [46] has documented both BVDV-2a and BVDV-1b circulating in Tunisia. To date, molecular sequencing data remain unavailable for Moroccan and Libyan BVDV isolates, underlining the need for targeted genetic surveillance in these regions.

This study is the first to report BVDV-1f strain in Algeria. Although Guidom et al. [28] previously identified BVDV-1a in Algerian cattle, our molecular analysis revealed that the isolate identified belongs to the 1f lineage, a genotype that has not been documented in the region before. Notably, these 1f strain shared high genetic similarity with isolates reported in Poland and Slovenia, suggesting a circulation of previously unknown strains. This subtype is most frequently reported in Germany and Slovenia, and is thought to be common in Europe [10]. Notably, Algeria imports substantial numbers of live cattle from Spain, where this subtype was reported by Eiras et al. [47]. These animal movements could account for the genetic proximity observed between our Algerian sequences and European isolates are a plausible route by which apparently healthy PI animals may have been introduced into the sampled herds. Nevertheless, phylogeographic conclusions remain tentative in the absence of denser sampling and higher-resolution genomic data.

As suggested by Köster et al. [46], wildlife may play a role in BVDV transmission. Wild boar (*Sus scrofa*), which are prevalent in northern forests [48] and have occasionally been reported as BVDV-positive in Serbia, appear unlikely to serve as major contributors to viral persistence in cattle; available evidence points more to sporadic spillover events than to sustained transmission. In contrast, small ruminants may constitute a more relevant reservoir. In Algeria, serological surveys have documented exposure to pestiviruses in sheep, with seroprevalence rates of 22.68% for BDV and 1.01% for BVDV [49]. At the flock level, nearly all flocks (98%) contained at least one seropositive animal, with a true within-flock prevalence estimated at 68.2% [50]. Taken together, multi-species herding practices [49], a scarcity of data on pestiviruses in wild ruminants [18] and the high adaptability of pestiviruses to multiple hosts [10] may help explain the relatively high proportion of BVDV-positive animals observed in our study.

Although no BVDV vaccines are currently in use in Algeria, antigenic variation between subtypes is still an important factor in the success of future eradication programmes. The recognition of BVDV-1f in this study emphasises the importance of accounting for this subtype when devising future control and vaccination strategies in Algeria.

The comparison to previous research affirms the need for epidemiological surveillance to better understand the development of BVDV and its effect on cattle health. In areas where BVDV is widespread and poorly controlled, transient infection is almost inevitable at some point in an animal’s life. The need for an integrated diagnostic strategy combining antibody ELISA for herd-level screening, antigen-detection ELISA on ear-notch samples for the identification PI animals, and RT-PCR for confirmatory detection of viral RNA. Importantly, diagnostic identification should be embedded within a comprehensive and context-specific BVDV management plan, including removal of PI animals, implementation of vaccination programs, reinforcement of biosecurity measures, regulation of animal movements, and continuous surveillance tailored to the targeted level of disease control or eradication.

The relatively small sample size may not accurately represent the epidemiological differences between regions or production systems. Future studies should involve a large sample size including non-cachectic animals, and tests for a wider range of pathogens related to cachexia in cattle.

## 5. Conclusions

This opportunistic case series documents a high proportion of BVDV-seropositive cattle and the genetic identification of BVDV-1f among cachectic cattle sampled in Tiaret province, Algeria. These results provide local baseline data and highlight the need for targeted, representative surveillance and longitudinal studies to identify PI animals and to assess the broader epidemiology of BVDV in Algeria. The detection of BVDV-1f in this province emphasizes that future control strategies and vaccine considerations should account for local subtype diversity.

## Figures and Tables

**Table 1:** Classification of tested animals by age and sex.

Herd ID	Number of Tested Animals	Sex Category		Age Category		
		Male	Female	<6 Months	6–12 Months	>12 Months
1	9	3	6	3	2	4
2	8	3	5	3	1	4
3	7	0	7	1	2	4
4	8	1	7	2	2	4
5	12	1	11	1	4	7
6	12	1	11	1	1	10
7	9	1	8	3	2	4
8	14	2	12	4	3	7
9	8	3	5	2	1	5
10	13	3	10	2	4	7
Total	100	18	82	22	22	56

**Table 2:** Proportion of BVDV antibody-positive animals among tested cattle, by age and sex.

Factors		Seropositive Animals (%)	95% CI (Wilson)
Sex	Female	70/74 (94.6%)	[83.8–96.3%]
	Male	18/18 (100%)	[82.4–100%]
Age	<6 months	18/19 (94.7%)	[75.4–99.1%]
	6–12 months	19/20 (95%)	[76.4–99.1%]
	>12 months	51/53 (96.2%)	[87.2– 99%]

## Data Availability

The original contributions presented in this study are included in the article/Supplementary Material. Further inquiries can be directed to the corresponding author.

## Supplementary Materials

The following supporting information can be downloaded at: https://www.mdpi.com/article/10.3390/vetsci12121193/s1, Figure S1. Agarose gel electrophoresis of RT-PCR products targeting the 5′UTR of BVDV; File S1 Matrix Output: Estimates of evolutionary divergence between Sequences; Supplementary Table S1: ELISA, RT-PCR and cell culture results.

## Abbreviations

The following abbreviations are used in this manuscript:

BVD	Bovine Viral Diarrhoea
BVDV	Bovine Viral Diarrhoea Virus
CP	Cytopathogenic
ELISA	Enzyme-Linked Immunosorbent Assay
IPMA	Immunoperoxidase Monolayer assay
NCP	Non cytopathogenic
RT-PCR	Reverse Transcription Polymerase Chain Reaction
Ct	Cycle Threshold
OD	Optical Density
ODNC	Optical Density of Negative Control
ODPC	Optical Density of Positive Control
