# Supplementary figures and images for: Detection of Bovine Viral Diarrhoea Virus in a Case Series of Clinically Cachectic Cattle from Tiaret, Algeria
Source: Vet Sci. 2025 Dec 12;12(12):1193. doi: 10.3390/vetsci12121193 (PMC12737618; doi:10.3390/vetsci12121193)

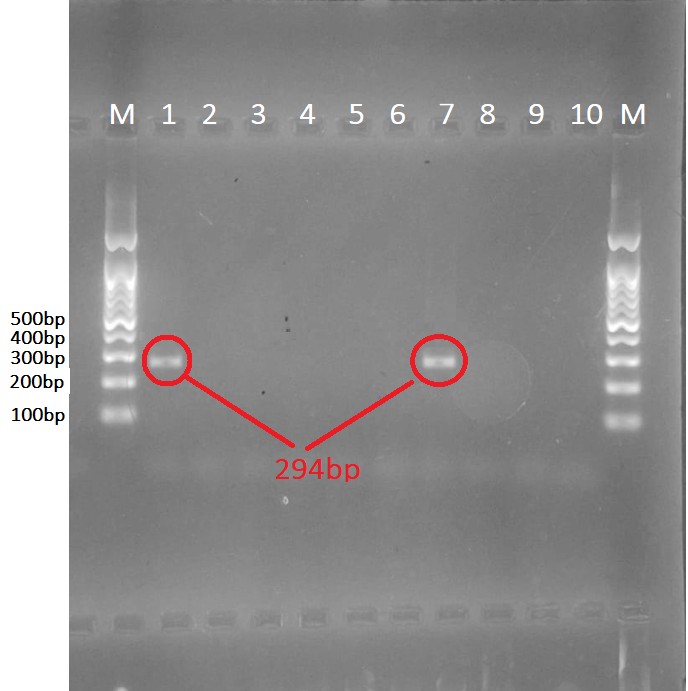

Supplement: Supplementary file 1 [file vetsci-12-01193-s001.zip › vetsci-3935976-supplementary/Figure S1 gel electr.jpg]
